# Stéatose hépatique aiguë gravidique avec une pyélonéphrite aiguë droite sur une grossesse gémellaire: une association très rare

**DOI:** 10.11604/pamj.2013.15.151.2373

**Published:** 2013-08-27

**Authors:** Zine el Abidine Benali, Karim Rachidi, Driss Omari

**Affiliations:** 1 Service d'anesthésie-réanimation, CHP Eddarak, Berkane, Maroc; 2 Service de maternité CHP Eddarak, Berkane, Maroc; 3 Service de Médecine Interne et des Maladies Cardiovasculaires, CHP Eddarak, Berkane, Maroc

**Keywords:** Grossesse gémellaire, pyélonéphrite aigue, stéatose hépatique, Twin pregnancy, acute pyelonephritis, hepatic steatosis

## Abstract

La Stéatose hépatique aiguë gravidique est une complication rare de la grossesse, et l'association avec la pyélonéphrite aiguë est encore plus rarissime survenant le plus souvent dans le troisième trimestre. Le diagnostic est affirmé par un faisceau d'argument clinique et biologique si non par l'histologie hépatique en dehors de trouble de la crase sanguine. Autrefois régulièrement mortelle, cette pathologie bénéficie actuellement d'un meilleur pronostic maternel et fœtal, du fait du diagnostic plus précoce, d'une délivrance rapide et du traitement symptomatique. Les auteurs ont jugé utile de rapporter une observation à travers d'un cas clinique d'une grossesse gémellaire associée à la pyélonéphrite aigue droite avec stéatose hépatique aiguë en milieu de réanimation.

## Introduction

La stéatose hépatique aiguë gravidique (SHAG) est une pathologie rare survenant durant le troisième trimestre de grossesse, découverte par Sheehan en 1940, elle représente la seule hépatopathie gravidique responsable d′une insuffisance hépatique aiguë. Dans la moitié des cas, des manifestations d′hypertension artérielle gravidique y sont associées [1]. La SHAG est une affection à ne pas méconnaître, car une prise en charge précoce améliore considérablement le pronostic fœtal et maternel. L′évolution est favorable sous traitement, mais au prix d′un accroissement des dépenses de santé et, parfois, d′importantes séquelles neurologiques. Nous rapportons ici un cas de SHAG sur une grossesse gémellaire associée à la pyélonéphrite aigue droite compliquée par une septicémie et syndrome de défaillance multiviscérale nécessitant des soins intensifs, en dépit de la cessation rapide de la grossesse.

## Patient et observation

**Figure 1:**
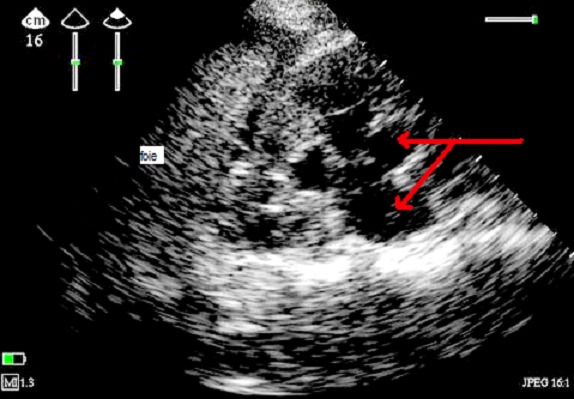
Image échographique mode 2D en coupe intercostale droite montrant une dilatation pyélocalicielle (flèche rouge) avec un épanchement minime intra péritonéal

**Figure 2:**
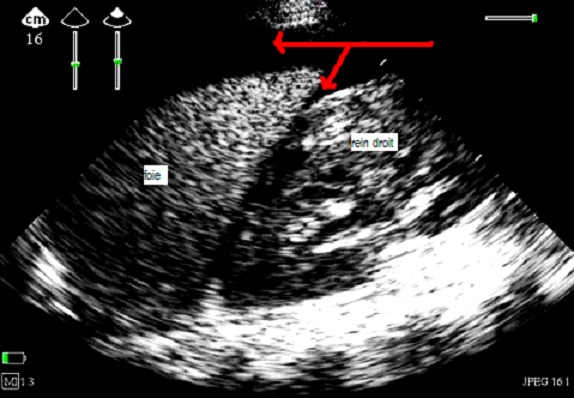
Image échographique mode 2D en coupe intercostale droite en deuxième jour de son hospitalisation montrant l'abondance de l’épanchement en péri hépatique et dans la poche de morrison (flèche rouge), la dilatation pyélocalicielle a régressée après son accouchement

Mme H., âgée de 26 ans, nullipare, primigeste, présentait depuis 5 jours des douleurs lombaires du coté droit, un ictère et des vomissements. La patiente était admise à la salle d'accouchement pour des contractions sur une grossesse gémellaire mal suivie, à 37 semaines d'aménorrhées selon la date de dernières règles. L'examen clinique trouvait, une patiente consciente, pression artérielle à 140–90 mmHg, fébrile à 39°C, une fréquence cardiaque à 100 battements par minute, un ictère cutanéomuqueux, des œdèmes des membres inférieurs. La patiente entrait spontanément en travail et l′examen obstétrical révélait une dilatation cervicale à 5 cm associée à des contractions utérines régulières. Les examens biologiques revalaient: un groupage O Rh positif, hémoglobine à 13 g/dl, une hyperleucocytose à 17000/mm3, les plaquettes à 120000/mm3, un taux de prothrombine bas à 10%, un taux de céphaline activé de 3× celui du témoin, Alanine Amino Transférase 10 × la normale, Aspartate Amino Transférase 11 × la normale, hyperbilirubinémie à 90 mg/l, créatinémie à 17 mg/l, urée à 0.37 g/l, glycémie à 0.54 g/l, les sérologies de l′hépatite A, B, C et E étaient négatifs et le diagnostic de SHAG était proposé. Après correction de la glycémie, et faire passer rapidement 7 unités de plasma frais congelé, avec 10 mg de vitamine K, le premier bébé était accouché par voie basse APGAR 8/10 de sexe masculin avec un poids de 2kg 500 mais le deuxième était décédé par l’ hémorragie intra amniotique. La maman était ensuite hospitalisée en réanimation 2 heures après l'accouchement, l’échographie au lit du malade montrait: une dilatation péylo-calicielle, le foie est d’échostructure et de taille normales, sans dilatation intra ni extra hépatique et sans visualisation d'un hématome sous capsulaire (Figure 1). Au deuxième jour, l'examen cyto-bactériologique des urines était positif avec présence d’ Escherichia coli, l’échographie abdominale montrait un épanchement péritonéal de grande abondance (Figure 2), avec 4 litres de liquide jaune citrin ponctionné sous échographie et l’ analyse cytobactériologique était négative biochimique type transsudat, l’échographie cardiaque montrait en plus un épanchement péricardique minime sans retentissement sur la fonction cardiaque,en plus la patiente présentait une insuffisance rénale aiguë oligoanurique jugulée par un traitement médical sans recours à la dialyse. Au septième jour elle présentait une détérioration de la conscience, un échantillon de sang artériel de gaz sur 4 L/min d′oxygène par un masque montrait un pH: 7,29, PCO 2: 32mmHg, PO2: 101mmHg, bicarbonate: 15.0 mEq/L, et le déficit de base standard de 9.6mEq/L, la patiente était intubée ventilée pendant cinq jours puis extubée, adressait au service de maternité le dix-septième jour.

## Discussion

L'ictère pendant la grossesse a de nombreuses causes, comme la cholestase intrahepatique, les hépatites virales, la lithiase biliaire, la pré-éclampsie avec ou sans HELLP syndrome (Hemolysis, Elevated Liver enzymes, Low Platelet count), et la SHGA. La cholestase intrahépatique de la grossesse peut présenter pendant le troisième trimestre, mais le prurit est le symptôme caractéristique et la bilirubinémie est rarement très élevée. La Lithiase biliaire peut se produire à n′importe quel moment au cours de la grossesse accompagnée de douleur de l'hypochondre droit, et l’échographie facilite le diagnostique. L′hépatite virale aiguë pendant la grossesse pose aussi un problème mais la sérologie aide et redresse le diagnostique. Toutes ces causes ont été écartées dans notre cas, sur la base de la présentation clinique, la biologie et l’échographie. Il faut savoir que le diagnostique différentiel le plus difficile de la SHAG est le HELLP syndrome car ils partagent de nombreuses similitudes dans les caractéristiques cliniques et biologiques. Les manifestations de la pré-éclampsie sont habituellement observées durant la deuxième moitié de la grossesse, alors que les symptômes de HELLP syndrome et la SHAG apparaissent fréquemment dans le troisième trimestre [2, 3]. L′incidence du HELLP syndrome est beaucoup plus élevé 1/5000 que celle de la SHAG 1/13000 [4]. Mais la coagulopathie sévère, l′ictère, l'encéphalopathie hépatique, l'ascite, l'hypoglycémie, et l’élévation significative des taux sériques de transaminases et de la bilirubine sont les principales caractéristiques de la SHAG comme dans notre cas clinique [5, 6]. Mais le diagnostique de certitude est la biopsie hépatique qui montre en évidence des microvésicules graisseuses au sein des hépatocytes mais cet examen n′est pas toujours possible vu les troubles de la crase sanguine chez notre patiente.

La physiopathologie de la SHAG reste encore discutée, mais les études ont révélé une incidence plus élevée chez les femmes qui ont une mutation génétique qui affecte leur voie mitochondriale d'oxydation des acides gras et qui portent un fœtus avec carence d'une longue chaîne-3- hydroxyacyl-coenzyme A déshydrogénase [2, 7].

L’élévation de la créatininémie est constante dans la SHAG et s′explique par le dysfonctionnement mitochondrial rénal. Lorsqu′elle existe, l′insuffisance rénale est de type fonctionnel. Le mécanisme physiopathologique de l'insuffisance rénale fonctionnelle est principalement une nécrose tubulaire aiguë avec oligurie secondaire à une hypovolémie elle-même secondaire aux troubles hémorragiques provoqués par l′insuffisance hépatocellulaire [8]. Plus de 60% des femmes enceintes développent l′insuffisance rénale aiguë, mais dans la plupart des cas l’évolution est favorable et sans avoir recours à la dialyse comme le cas de notre patiente [9]. La fièvre, les douleurs lombaires, la dilatation pyélo-calicielle, et la présence d’ Escherichia coli à l'ECBU évoquent une pyélonéphrite aiguë, bien qu’ elle affecte seulement 1-2% des femmes enceintes [10], cette association avec la SHAG est encore plus rare en aggravant le pronostique maternel et fœtal.

Le traitement consiste l′interruption de grossesse, associée à une correction symptomatique des désordres biologiques et à prévenir les complications maternelles. L′accouchement par voie basse est préférable en cas de troubles majeurs de la coagulation et/ou de mort fœtale in utero comme chez notre patiente. Avant 1980, les taux de mortalité maternelle et fœtale étaient environ de 75% et les principales causes étaient l′œdème cérébral, l'hémorragie gastro-intestinale, l'insuffisance rénale, la coagulopathie, la pancréatite, et la septicémie. La mortalité a été réduite à moins de 10% à l′heure actuelle [11] en raison d′une meilleure reconnaissance et prise en charge multidisciplinaire.

## Conclusion

La stéatose hépatique aiguë gravidique reste une maladie grave pour la mère et le nouveau-né. La cause est généralement encore inconnue, rendant le diagnostic et la prévention difficile, Le pronostic est lié à la prise en charge précoce. Le traitement est l'interruption de grossesse, associée à une correction symptomatique des désordres cliniques et biologiques. Il est recommandé que les patientes qui sont gravement malades au moment de l'accouchement, et qui développent des complications, doivent être gérées en milieu de la réanimation.
